# A Comprehensive Review of Hip Fractures in Sub-Saharan Africa: Contextual Challenges, Outcomes, and Pathways to Improved Care

**DOI:** 10.7759/cureus.97653

**Published:** 2025-11-24

**Authors:** Ezeokoye Maria, Obed Amoako-Adjei

**Affiliations:** 1 Trauma and Orthopaedics, Mid Yorkshire NHS Teaching Trust, Wakefield, GBR; 2 General Surgery/Trauma and Orthopaedics, Mid Yorkshire NHS Teaching Trust, Wakefield, GBR

**Keywords:** hip fracture, management of hip fracture, neck of femur fracture, outcomes of hip fracture, sub-saharan africa

## Abstract

Neck of femur fractures remain a major cause of orthopaedic morbidity and mortality globally, with an increasing burden in Sub-Saharan Africa (SSA) as populations age and trauma persists among younger groups. This review summarises the epidemiology, pathophysiology, management, and system-level challenges associated with hip fractures across SSA.

Through a narrative synthesis of 37 regionally relevant studies published between January 1995 and May 2025, we explore how systemic resource limitations, delayed presentation, comorbidities, and workforce shortages shape clinical outcomes.

Despite advances in surgical training and capacity-building, the region continues to face unique constraints, including limited access to operative fixation, delayed presentation, workforce shortages, and socioeconomic barriers to care.

Emerging regional data highlight a dual burden: fragility fractures in the elderly alongside high-energy trauma in younger adults. Mortality rates remain substantially higher than that in high-income countries, reflecting both systemic and peri-operative challenges.

Efforts to integrate hip-fracture care into essential surgical packages and strengthen rehabilitation services are ongoing but at a slow pace and in only a few sub-regions. Investment in prevention, early surgical intervention, and workforce development will be critical to improving outcomes.

## Introduction and background

Neck of femur (NOF) fractures are a major cause of morbidity, functional disability, and mortality worldwide, particularly among the elderly. Globally, the annual incidence is projected to increase from 1.66 million in 1990 to more than six million by 2050, driven by the ageing population and increasing prevalence of osteoporosis [1]. Although once considered a problem confined to high-income countries, recent evidence suggests that low- and middle-income countries (LMICs), especially in Sub-Saharan Africa (SSA), are witnessing a sharp rise in these fractures [2,3].

NOF fractures, as mentioned in this review, include both intracapsular and closely related extracapsular fractures of the proximal femur, typically occurring either as low-energy fragility injuries in older adults or high-energy trauma in younger patients. Fragility fractures refer to low-impact injuries associated with osteoporosis and age-related bone loss and account for a substantial proportion of the rising NOF fracture burden in SSA.

This trend in SSA reflects complex demographic and epidemiologic transitions. While infectious diseases continue to impose a heavy health burden, longer life expectancy and urbanisation have increased the prevalence of non-communicable diseases such as diabetes, hypertension, and osteoporosis, which contribute to fragility fractures. At the same time, high-energy trauma from road traffic accidents (RTAs) continues to cause NOF fractures in younger populations. As a result, the region faces a mixed burden of fragility and high-energy fractures, each requiring different approaches to management [4,5].

In high-income countries, early surgical fixation or arthroplasty, ideally within 24-48 hours, combined with perioperative optimisation and multidisciplinary rehabilitation, significantly reduces mortality and improves functional outcomes [6]. However, these best-practice standards are difficult to achieve in most SSA settings due to delays in presentation, diagnostic limitations, implant shortages, inadequate operating theatre availability, and financial barriers [7,8]. In many parts of SSA, rehabilitation and social reintegration are underdeveloped, leading to chronic immobility, dependency, and socioeconomic hardship for patients and families [9]. 

Despite an increasing number of regional studies, current evidence from the region remains sparse and fragmented and is often limited to single-centre retrospective studies. There is a lack of comprehensive synthesis of research that addresses epidemiology, management pathways and outcomes. There is no consensual framework in existence currently that guides context-specific management pathways.

The purpose of this review is to describe the epidemiological patterns and determinants of NOF fractures in SSA, examine current management practices, patient outcomes and system barriers to optimal care and explore prevention strategies, economic implications and policy priorities tailored in the context of SSA.

## Review

Methodology

This narrative review was conducted using the literature retrieved from PubMed, Google Scholar, and African Journals Online (AJOL). The search covered publications between January 1995 and May 2025. Search terms included combinations of “neck of femur fracture,” “hip fracture,” “sub-Saharan Africa,” “hemiarthroplasty,” “dynamic hip screw,” “management,” “outcomes,” and “mortality.”

Studies were included if they: were conducted in SSA; involved adult patients (≥18 years); addressed epidemiology and clinical management; addressed outcomes or system-level challenges; were case series with more than 10 patients, retrospective studies, cohort studies, systematic reviews and meta-analyses and randomised controlled trials; and were case reports. Case series with <10 patients, and pediatric studies were excluded. Reference lists of included papers were manually screened to identify additional studies.

The initial search resulted in over 70 papers; after applying the exclusion criteria, this was narrowed down to 37 based on our criteria.

In total, 37 eligible studies were analysed and narratively synthesised. Data were extracted on study design, population demographics, fracture classification, management approach, complications, and mortality.

The included studies varied substantially in design (retrospective vs. prospective), sample size, management approaches, and reported outcome measures. These differences prevented meaningful pooling of data and therefore made meta-analysis inappropriate.

While formal meta-analysis was not feasible due to heterogeneity, common trends were identified and thematically analysed to produce a contextualised understanding of management challenges and opportunities in SSA. Limitations of this narrative review include the scarcity of published data from many countries within SSA and the predominance of small, retrospective, single-centre studies, which may introduce selection bias, incomplete follow-up, and reporting variability.

The quality of this narrative review was evaluated using the SANRA (Scale for the Assessment of Narrative Review Articles) framework, which assesses reviews across six domains: scope, accuracy, relevance, novelty, applicability, and scientific reasoning/presentation. Each domain was scored from 0 to 2, with a maximum possible total score of 12. This review scored 2 for scope, as it comprehensively addressed both elderly fragility fractures and high-energy trauma in younger adults; 2 for accuracy, reflecting reliable synthesis aligned with cited regional data; 2 for relevance, given its importance for clinical practice, health policy, and orthopedic care in SSA; 2 for novelty, for providing new regional insights and highlighting the dual-burden fracture epidemiology; 2 for applicability, due to actionable recommendations for clinicians and health systems; and 1 for scientific reasoning and presentation, acknowledging clear structure and logical synthesis despite the absence of meta-analytic precision. Overall, the review achieved a total SANRA score of 11 out of 12, demonstrating high quality, rigour, and relevance in the context of SSA.

Epidemiology

Hip fractures are increasingly common across SSA, reflecting a growing ageing population and a rise in osteoporosis-related fragility fractures. The burden of disease is likely underestimated due to underreporting and limited orthopaedic surveillance systems [1,2]. Globally, hip fractures disproportionately affect women, particularly postmenopausal females, due to hormonal and bone density changes. This gender pattern is mirrored in SSA, though the region also reports a substantial proportion of younger male patients affected by high-energy trauma [9-11].

A multi-centre South African study reported that the incidence of hip fractures among Black South Africans has steadily increased over the last two decades, closing the gap with historically higher-risk White and Asian populations [3]. Although multiple studies describe higher fracture burdens in urban populations than in rural areas, precise population-based incidence data remain unavailable. However, hospital-based data from Ghana, Botswana, and South Africa suggest that urban centres report substantially greater numbers of hip fracture admissions, largely reflecting population density and care accessibility [2,3,8]. In contrast, in Somalia and Nigeria, a significant share of NOF fractures occur in men under 50, often due to RTAs or occupational injuries [9-11].

These findings highlight a dual burden: fragility fractures in elderly women and high-energy fractures in young men. This epidemiologic reality complicates regional management strategies, as treatment approaches and rehabilitation needs differ substantially between the two groups.

**Figure 1:**
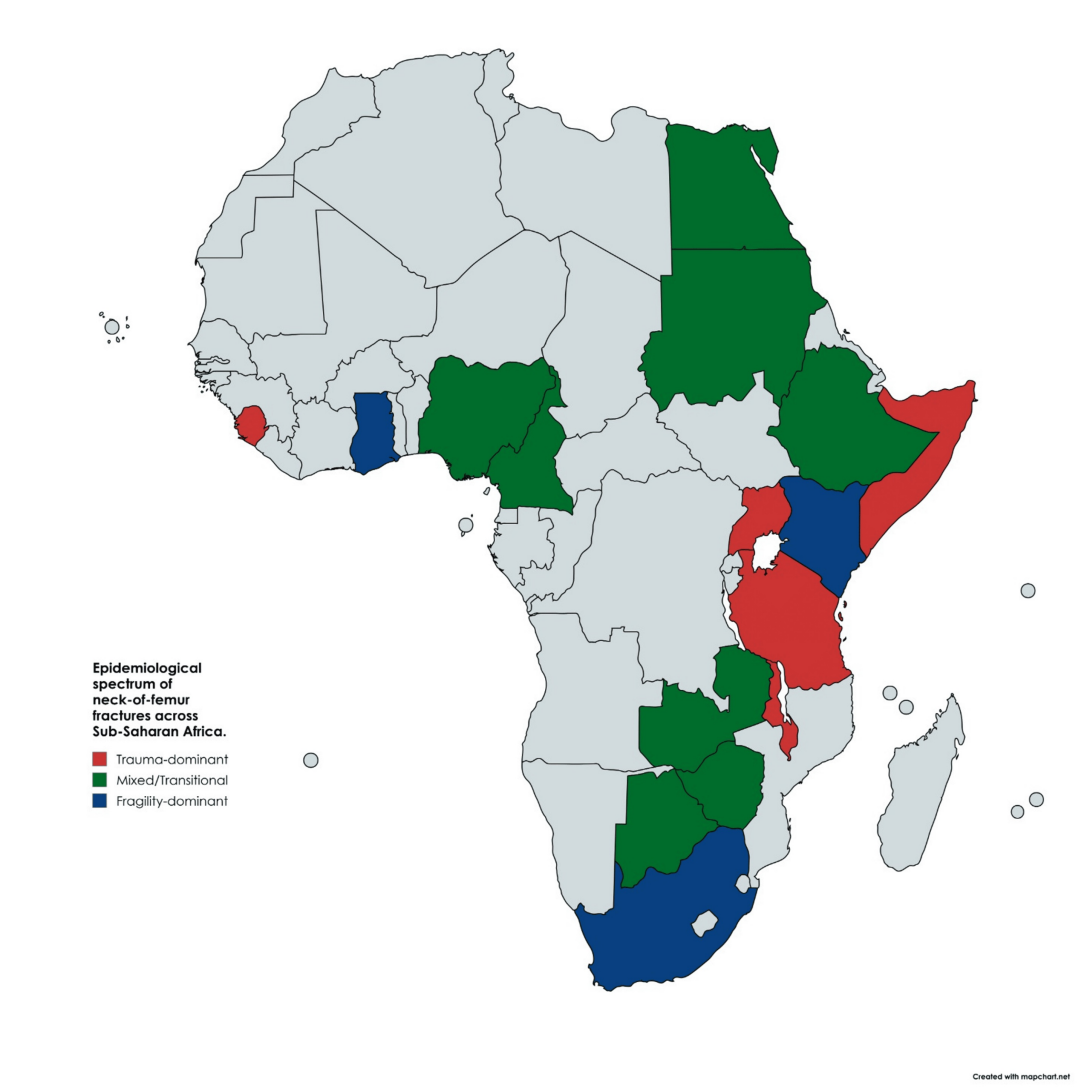
Epidemiological Spectrum of Neck-of-Femur Fractures in Sub-Saharan Africa The map illustrates the dual burden of hip fractures, with trauma-related injuries (red) predominating in low-income rural regions, a transitional pattern (green) in urbanising countries, and fragility fractures (blue) increasing in more developed or ageing populations [1,2,8-11] Created by authors using mapchart

Figure 1 demonstrates the epidemiological pattern of hip fractures in SSA.

Anatomy, biomechanics and pathophysiology

The blood supply of the femoral head primarily arises from the medial and lateral femoral circumflex arteries, which form the retinacular vessels that traverse the femoral neck. Disruption of these vessels during a displaced intracapsular fracture compromises perfusion, increasing the risk of developing avascular necrosis (AVN) and non-union. In elderly patients, microvascular disease and osteoporosis further impair bone healing capacity [12]. The femoral neck in biomechanical terms can be likened to a cantilever beam, which transmits weight and forces of abduction through its trabecule. Advanced age comes with thinning of the cortices and loss of connectivity in the trabecular bone. This increases the stress concentration, making it easy for less energy trauma, such as falls from standing height, to cause complete neck of femur fractures [13].

In high-energy trauma, the hip is subjected to axial and rotational loads, which tends to produce a vertical fracture pattern with high shear forces as seen in Garden Type III to IV [14].

Bone healing is often impaired in elderly, malnourished and immunocompromised patients such as Human immunodeficiency virus (HIV) positive patients. This is due to impaired osteoblastic activity and chronic inflammation. Prolonged immobilisation, common in SSA due to late presentation, and delayed surgery further reduce local perfusion and increase the risk of thromboembolic events [15,16]. Understanding these biomechanical and biological factors is crucial for contextualising the high non-union and AVN rates reported across the region.

Current management approaches

Elderly Patients

For elderly patients with displaced intracapsular fractures, hemiarthroplasty remains the most common surgical option in SSA [6]. Total hip arthroplasty is reserved for active, independent patients where resources permit. Studies from South Africa and Kenya demonstrate that hemiarthroplasty offers acceptable short-term outcomes and improved mobility compared to conservative treatment, though long-term follow-up remains limited [5,7,8].

**Figure 2:**
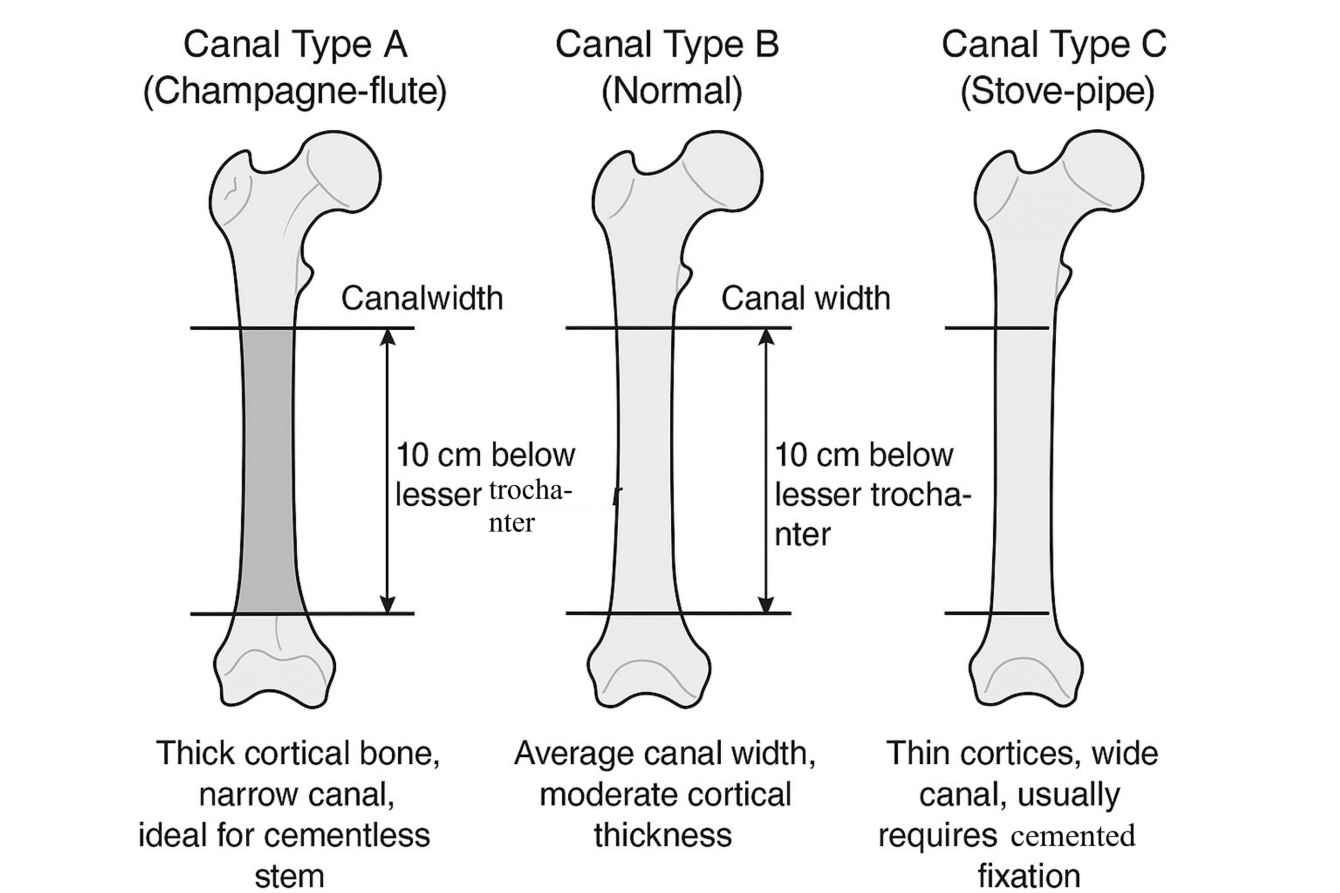
Dorr Classification of the Proximal Femoral Canal Created by authors using Canva based on the text from [18]

Implant selection, particularly cemented versus cementless stems, is influenced by bone quality, degree of osteopenia, and the morphology of the proximal femur. The Dorr classification (Figure 2) helps guide this decision: Dorr A and B femurs, with preserved cortical thickness, are more suitable for cementless stems, whereas Dorr C femurs, characterised by severe osteopenia and a wide canal, typically favour cemented fixation [17,18]. In some settings, such as Egypt, cementless implants are widely used due to surgeon preference and reported reductions in early complications [19].

Despite these advances, mortality remains high, with 30-day rates between 7 and 10 % and one-year mortality reaching up to 40 % [5,20]. Trochanteric fractures Arbeitsgemeinschaft für Osteosynthesefragen/Orthopaedic Trauma Association (AO/OTA) 31A are often treated with a cephalomedullary nail or a dynamic hip screw. Cephalomedullary nail tends to be the favoured option when there is a subtrochanteric extension [21]. In Egypt, the use of short gamma nails has been reported to offer shorter operative times, lower radiation exposure, and less intra-operative blood loss, with no significant difference in clinical or radiological outcomes [22].

Younger Adults

In patients under 60 years, internal fixation using multiple cannulated screws or a dynamic hip screw (DHS) is the treatment of choice to preserve the femoral head [9,10]. However, delayed presentation and limited imaging contribute to technical challenges and increased rates of non-union (7.4-35%) and avascular necrosis (11.5-45%) [23]. Despite these risks, surgical fixation remains superior to conservative care, offering functional preservation when performed early.

For DHS, tip apex distance has been shown to be the most important predictive factor for implant failure; other factors such as position of the lag screw, fracture reduction and patient’s bone quality and co-morbidities also affect this [24]. Other centres in Kenya for example favoured the use of Intramedullary nail fixation over DHS for intertrochanteric fractures [25].

Conservative Management

Conservative management, largely phased out in high-income settings, remains a reality in many SSA hospitals when implants, theatre slots, or surgeon availability are constrained [26,27]. Prolonged traction or bed rest is associated with significant morbidity, including pressure ulcers, DVT, and pneumonia.

However, strictly selected stable fracture patterns, particularly AO/OTA 31A1.2 pertrochanteric fractures, can unite successfully when managed within structured conservative protocols that include close radiographic monitoring and conversion to surgery if displacement develops [26]. The criteria for non-operative care used include: minimally displaced, mechanically stable fracture pattern, reliable follow-up, absence of medical contraindications to prolonged immobilisation and lack of surgical resources or patient inability to afford implants.

Decision Tools

The Sernbo score, validated in South Africa, provides a pragmatic approach to stratifying patients for arthroplasty types based on age, comorbidities, and mobility. This locally validated scoring system has demonstrated comparable predictive value to international indices and supports context-appropriate decision-making [28]. In the South African study, patients with a Sernbo score <15 experienced a significantly higher mortality rate (15.4%) compared to those with scores ≥ 15 (8.9%), indicating lower Sernbo scores may predict poor outcomes.

The Sernbo score incorporates four parameters: age, mobility, living circumstances, and mental status, with each scored from 0 to 5.

Challenges in management

Delayed Surgical Intervention

Timely surgical intervention is a cornerstone of hip fracture care. However, in SSA, surgery within 72 hours remains rare [29]. Average time from injury to operation ranges from 7-19 days, often exceeding two weeks [6,28]. Causes include delayed hospital arrival, limited theatre capacity, implant unavailability, preoperative optimisation delays, and financial barriers. Delayed health-seeking behaviour of patients who have sustained injury played a significant role in delay to treatment in studies in urban centres in South Africa and Malawi, the average time it took for initial presentation was five days, which is suspected to be even worse in rural areas [29,30]. One of the peculiar reasons recognised for this delay is initial attendance at traditional care centres for trial of traditional bone setting methods. Other studies report delayed inter-hospital transfers, which is primarily driven by inadequate ambulance transportation systems and financial barriers. The definition for early surgery in the context of such low-resource settings was redefined in some settings as surgery within seven days [31].

Workforce and Infrastructure

SSA faces a severe shortage of orthopaedic surgeons, anaesthetists, and perioperative staff. Theatre density averages 1-2 per 100,000 population compared to over 14 in high-income countries [32]. Limited access to imaging, implants, and sterile equipment compounds delays and restricts surgical choices [33,34].

The density of orthopaedic surgeons in SSA remains critically low compared to developed countries, highlighting a major workforce challenge in the region. In countries such as Malawi and Nigeria, there are as few as 0.03 and 0.22 orthopaedic surgeons per 100,000 population [35-37], respectively, while even in South Africa, which has the highest regional density, there are only 1.63 per 100,000, with most specialists concentrated in urban centres [38]. By contrast, developed countries such as the United States, the United Kingdom, and Germany have between 8 and 15 orthopaedic surgeons per 100,000 population [39,40]. This stark disparity, often more than 20-fold, directly contributes to delayed surgical care, limited access to specialised interventions, and poorer patient outcomes, particularly in rural and underserved areas. The severe shortage underscores the urgent need for workforce strengthening, task-sharing models, regional training programs, and equitable distribution of specialists to improve orthopaedic care across Sub-Saharan Africa.

Comorbidities

High rates of HIV, malnutrition, anaemia, and uncontrolled chronic diseases such as diabetes and hypertension present additional peri-operative challenges in SSA. These conditions significantly influence postoperative recovery, wound healing, and overall mortality.

Studies from Malawi and Tanzania show that, with appropriate optimization, including nutritional support, antiretroviral therapy, and peri-operative antibiotic prophylaxis, HIV positive patients can have outcomes comparable to HIV negative patients. This is more particular when cluster of differentiation 4 (CD4) counts are adequate with reduced viral load [34,41].

Anaemia, which is quite prevalent at presentation in SSA due to chronic nutritional deficiencies, has been shown to correlate with increased transfusion requirement, prolonged hospital stay and poor post-operative mobility and outcomes [42]. Optimising haemoglobin prior to surgery especially for pre-operative levels less than 10g/dl significantly improves outcomes, especially among the elderly and frail population

Within the region, malnutrition has been shown to affect up to a third of patients with hip fractures. This significantly increases post-operative infection risks, risks of pressure sores due to delayed mobility recovery. Integrated nutritional assessment in pre-operative protocols is not routinely practised [15]. Collectively, these comorbidities highlight the importance of perioperative optimisation and multidisciplinary management, particularly in resource-limited hospitals where laboratory and nutritional support may be minimal.

Preoperative Care

Evaluation of patients with hip fractures across SSA typically involves a focused history, physical examination, and plain radiography, with anteroposterior and lateral views of the pelvis and affected hip forming the diagnostic standard. Advanced imaging modalities such as computed tomography or magnetic resonance imaging are rarely available or used routinely, except in tertiary referral centres, largely due to cost and limited access [19,31].

Anaesthetic management varies across institutions but generally favours spinal or epidural anaesthesia, which are considered the most dependable and resource-appropriate method in low- and middle-income settings. These techniques are cost-effective, have lower pulmonary complication rates and relatively require less monitoring infrastructure both intra-operatively and post-operatively when compared to general anaesthesia [19].

However, the ultimate choice often depends on expertise availability and patient comorbidities, with general anaesthesia still used in complex cases or those with contraindications to regional anaesthesia [43].

Pre-operative optimisation remains a major determinant of postoperative outcomes. Many patients in SSA present late, often several days after fracture. This consequently leads to prolonged immobilisation, dehydration, anaemia, and nutritional depletion. For example, in a South African cohort, 69.5 % were anaemic on admission, and the median time to surgery was 19 days [42]. Routine laboratory assessment (haemoglobin, creatinine, electrolytes, HIV status) is variably performed depending on facility resources, and multidisciplinary pre-operative input is often not available except in large tertiary centres [44].

Due to prolonged hospital stays prior to surgery, many centres implement pharmacological thromboprophylaxis using low-molecular-weight heparin or unfractionated heparin, which is typically discontinued 24 hours before surgery to minimise bleeding risk [17].

Antibiotic prophylaxis is a cornerstone of infection prevention. In most SSA centres, a single preoperative dose of a first-generation cephalosporin is administered at induction. However, some institutions extend antibiotic use for up to five days postoperatively, particularly in arthroplasty cases to minimise risk of prosthetic joint infections due to concerns about wound contamination [5,8,20,42].

Socioeconomic and Cultural Barriers

In most SSA countries, healthcare is financed predominantly through out-of-pocket payments, with minimal insurance coverage for orthopaedic services. This financing model imposes a major barrier to timely fracture surgery. The high cost of implants and surgical fees often forces patients and families to delay or forego operative management altogether, with preference for conservative management, leading to preventable disability and mortality [43-45].

In Kenya and Tanzania, even where surgery is available, the cost of a single hip fixation or hemiarthroplasty can equal 6-8 months of median household income, excluding physiotherapy and transportation expenses [45,46].

The economic burden on caregivers further compounds these challenges. A Tanzanian study reported that informal caregivers lost approximately 10 % of their annual income while providing postoperative support and rehabilitation for hip-fracture patients [46]. In low-income households, such indirect costs can be unbearable, particularly when recovery delays over months.

Cultural beliefs also play a major role in delaying care. Trust in traditional bone-setters remains widespread, particularly in rural communities where access to hospital-based services is limited. Many patients initially seek help from bone-setters, only presenting to hospitals after complications [29,47].

Effective patient counselling is an essential component of preoperative care. Communication barriers, including language differences and low literacy levels, can delay decision-making and reduce adherence to treatment plans. Involving family members and caregivers in preoperative and postoperative discussions has been shown to improve understanding and postoperative compliance [44].

Ultimately, socioeconomic hardship, cultural beliefs, and inadequate patient communication compound to result in delayed presentation and poor outcomes. Addressing these factors requires community-level education, integration of traditional and formal healthcare providers, improved financial protection schemes and better healthcare financing systems to mitigate this.

Rehabilitation Deficits

Postoperative rehabilitation, though a key component of recovery, remains one of the most under-resourced aspects of care in SSA. Most physiotherapy centres are located in urban tertiary hospitals, leaving a significant percentage of the population without access to structured rehabilitation [43,44]. This deficiency contributes to prolonged immobility, muscle wasting and even worse socioeconomic dependency. In Nigeria, some centres initiate quadriceps-strengthening exercises as early as postoperative day 1-2, often under direct physiotherapist supervision [31]. Early mobilisation is associated with faster recovery of independence, shorter hospital stays, and reduced thromboembolic risk.

In Togo, approximately two-thirds of patients treated with internal fixation are permitted protected weight bearing with canes from day one post-operatively, while the remainder-typically those with unstable fractures or poor fixation quality-begin partial or full weight bearing after about six weeks [48]. These findings demonstrate encouraging adaptation of early mobilisation principles even in resource-constrained settings.

The World Health Organisation’s (WHO) Rehabilitation 2030 framework notes that many SSA countries face severe rehabilitation workforce shortages [49]. Integration of physiotherapy and rehabilitation into national essential health service packages remains limited, though pilot programs in Tanzania and Ghana have demonstrated that incorporating trained rehabilitation assistants can be both feasible and cost-effective [50].

Addressing these deficits requires investment in rehabilitation workforce training, community-based programs, and inclusion of physiotherapy in universal health coverage schemes.

Outcomes and mortality

Medium-term and functional outcomes following hip fracture management in SSA have been assessed using tools such as the Modified Harris Hip Score and the Postel and Merle d’Aubigné hip function score, with approximately two-thirds of patients reporting good to excellent outcomes across various regional studies [25,48,51]. These findings suggest that, despite systemic constraints, satisfactory recovery is achievable when surgery and rehabilitation are timely.

Physical autonomy of elderly patients, assessed using the Katz Index of Independence in Activities of Daily Living, shows that only about one-fifth of patients regain their pre-injury level of autonomy one year post-surgery. Preoperative anaemia and an ASA classification ≥ 2 significantly reduced the likelihood of full recovery, while delay to surgery and late hospital admission were both independently associated with deterioration in post-operative autonomy [52].

Radiological outcomes tend to be satisfactory, with up to 70 % of cases achieving acceptable alignment and healing following intramedullary nailing in Togo [49]. Mortality, however, remains substantially higher than global averages: reported one-year mortality rates range between 30-40 % in SSA, compared with 15-20 % in high-income settings. The primary drivers of poor outcomes are surgical delay, comorbid conditions, lack of rehabilitation, and hospital-acquired complications [5,20,42,52].

Functional recovery remains suboptimal. Only about 22 % of elderly patients regain full pre-injury independence after surgery, and up to one-third remain non-ambulant at one year [52]. Nonetheless, early surgery is associated with markedly improved recovery: in studies from South Africa and Ghana, nearly 70 % of surgically treated patients regained independent mobility within three months [8,20].

Functional outcomes, measured by the Harris Hip Score, show steady improvement over time, with approximately 76 % achieving good-to-excellent results by six months post-surgery [8,19]. The timing of surgery and access to rehabilitation are consistently stronger predictors of outcomes than implant choice or surgical technique.

Postoperative Complications

The spectrum of postoperative complications varies widely across SSA. Studies from Egypt and Nigeria report common issues such as dislocation, deep infection, and revision surgery among arthroplasty patients, affecting between 2 and 5 % of cases [8,19]. The likelihood of complications increases with the number of comorbidities, especially diabetes, cardiovascular disease, and malnutrition [42].

Infection rates following internal fixation range between 6-15 %, while non-union and AVN occur in 10-20 % of femoral neck fractures, reflecting the impact of delayed fixation and vascular compromise [9,11,34].

Quality of life following arthroplasty has been evaluated using the EuroQol health-related quality of life questionnaire and EuroQol visual analogue scale (EQ-5D and EQ-VAS) tools. In Nigeria, over two-thirds of elderly patients who underwent arthroplasty reported good postoperative quality of life at six months [17].

Overall, these findings highlight the importance of early intervention, comorbidity optimisation, and postoperative rehabilitation. Although mortality and functional limitations remain higher than global averages, prompt surgery and structured rehabilitation can produce outcomes approaching international benchmarks.

Regional Disparities

Marked regional disparities exist in NOF fracture management across Sub-Saharan Africa. Urban tertiary centres in South Africa, Kenya, Egypt and Nigeria routinely offer arthroplasty and internal fixation, whereas rural hospitals in Malawi, Somalia, and Sierra Leone still rely largely on conservative treatment due to limited surgical infrastructure [17,20,21,31]. This highlights the need for decentralised orthopaedic services and the standardisation of essential trauma-care protocols.

Regional initiatives such as the College of Surgeons of East, Central, and Southern Africa (COSECSA) training programme and AO Alliance have expanded orthopaedic capacity through harmonised education and fellowship schemes, yet retention of skilled surgeons remains a persistent challenge owing to emigration and uneven workforce distribution [53].

Discussion

The management of NOF fractures in SSA reflects the broader challenges of surgical system strengthening in LMICs. Despite the increasing burden of these injuries, access to timely, high-quality care remains severely limited, and this continues to translate into poor outcomes, prolonged disability, and high mortality.

Systemic Barriers

Key barriers include insufficient theatre capacity, limited implant supply, financial constraints, and logistical delays. Most public hospitals cannot guarantee timely access to implants such as DHS or prostheses. Strengthening supply chains and establishing regional implant banks could mitigate these barriers and support more consistent surgical care.

Human Resource Gaps

The scarcity of trained orthopaedic surgeons is critical. Most SSA countries have fewer than two orthopedic surgeons per 100,000 people, compared with 10-20 per 100,000 in high-income countries [35-37]. Workforce shortages are compounded by uneven distribution, with specialists concentrated in urban areas. Task-sharing models involving general surgeons or advanced clinical officers in Malawi and Uganda have shown promising outcomes [54,55], highlighting the potential of context-specific solutions.

Economic and Health-System Implications

Hip-fracture care imposes a major economic burden on both patients and health systems. In Tanzania, informal caregivers lose approximately 10% of their annual income, and the average cost of hemiarthroplasty equals 6-8 months of median household income [46]. Out-of-pocket financing remains dominant [45], meaning treatment choices are often dictated by affordability rather than clinical need. Expanding local implant manufacturing and adopting lower-cost technologies may reduce dependency on imported implants in the future [56].

From a broader health-system perspective, untreated fractures contribute to long-term disability and productivity loss, undermining progress toward Sustainable Development Goal (SDG) 3.8 on universal health coverage. Integrating hip-fracture care into Essential Surgical Packages, as recommended by WHO, could reduce catastrophic expenditure and improve equity [57].

Rehabilitation and Continuum of Care

Postoperative rehabilitation remains severely under-resourced across the region. Most patients do not have access to structured physiotherapy outside urban tertiary centres. Community-based rehabilitation programmes, caregiver training, and integration of physiotherapy into primary care could improve functional recovery and reduce long-term dependency.

Public Health and Prevention

Preventive medicine efforts remain limited. High prevalence of Vitamin D deficiency among older adults suggests that supplementation programmes may reduce fragility-fracture risk. Falls prevention strategies such as home safety modifications and community exercise programmes are critical, especially since almost a quarter of falls occur among community-dwelling older adults [58,59]. In younger populations, improved road-safety enforcement, helmet use, and workplace injury prevention remain essential.

Research and Data Systems

A major challenge remains the absence of national fracture registries. Establishing multicenter databases would enable regional benchmarking and assessment of outcomes. Compared with high-income settings, where median time-to-surgery is <48 hours and one-year mortality is <20%, SSA patients frequently wait 7-19 days and experience one-year mortality of 30-40% [29,42]. These disparities reinforce the systemic nature of current limitations.

To contextualize these contrasts more clearly, Table 1 provides a summary comparison of epidemiological patterns, treatment timelines, and outcomes between SSA and high-income countries, highlighting key areas where gaps remain.

**Table 1:** Comparative Patterns of Neck of Femur Fractures in High-Income Countries (HICs) vs Sub-Saharan Africa (SSA) Table compiled by authors based on [1,2,5,9,11,23,26,29,44]

Characteristic	HICs	SSA
Age Distribution	Predominantly elderly (≥65 yrs)	Bimodal: elderly (fragility fractures) + younger adults (high-energy trauma)
Sex Distribution	Female > Male (osteoporotic fractures)	Elderly: Female > Male; Young: Male > Female (trauma-related)
Mechanism of Injury	Low-energy falls in elderly	Low-energy falls in elderly; high-energy trauma (RTAs, falls from height) in younger adults
Time to Hospital Presentation	Within hours	Often delayed (days–weeks), especially in rural settings
Time to Surgery	<48 hours (standard of care)	Average 7–19 days after injury
Common Treatment Modalities	Arthroplasty for elderly, fixation for younger adults	Internal fixation widely used; arthroplasty limited by cost and surgeon availability; non-operative management still common
Post-operative Rehabilitation	Structured, multidisciplinary	Sparse; limited or absent in rural areas
Mortality (1-year)	15–20%	30–40%

The expansion of COSECSA and partnerships such as the AO Alliance has improved regional training capacity, although specialist retention and equitable distribution continue to pose challenges. Incorporating orthopaedic care into national health-financing schemes and creating regional hip-fracture registries will further support quality improvement.

## Conclusions

NOF fractures represent a growing orthopaedic and public health challenge in SSA, driven by population ageing and persistent high-energy trauma. This review highlights the region’s dual epidemiologic burden, the wide variation in management practices, and the persistently high mortality that is largely influenced by delayed presentation, limited surgical capacity, financial constraints, and inadequate rehabilitation. Despite these constraints, regional data demonstrate that when timely surgery and structured postoperative care are achievable, patient outcomes can approach global standards.

Closing the gap will require strengthening core components of the surgical ecosystem: expanding workforce capacity, improving implant availability, integrating rehabilitation into routine care, and addressing the financial barriers that limit access to operative treatment. Prevention strategies, enhanced data systems, and context-appropriate management pathways will further support more equitable and effective care. Ultimately, improving hip-fracture management in SSA is both a clinical necessity and a marker of broader health-system resilience and commitment to healthy ageing.
